# Current status of family medicine in Kenya; family physicians’ perception of their role

**DOI:** 10.4102/phcfm.v12i1.2404

**Published:** 2020-09-21

**Authors:** Kaja Momanyi, Geert-Jan Dinant, Marlieke Bouwmans, Simone Jaarsma, Patrick Chege

**Affiliations:** 1 Department of Global Health, Faculty of Health, Medicine and Life Sciences, Maastricht University, Maastricht, the Netherlands; 2 Department of Family Medicine, Faculty of Health, Medicine and Life Sciences, Maastricht University, Maastricht, the Netherlands; 3 School of Health Professions Education Collaborates, Faculty of Health, Medicine and Life Sciences, Maastricht University, Maastricht, the Netherlands; 4 Department of Family Medicine, School of Medicine, Moi University, Eldoret, Kenya

**Keywords:** family medicine, primary healthcare, rural health, family practitioners, Kenya

## Abstract

**Background:**

Family medicine (FM) was introduced in Kenya in 2005. Up to date (August 2019), 42 family physicians have graduated from Kenyan Universities.

**Aim:**

The aim of the study was to establish the current state of FM in Kenya and identify areas for more research and actions to support and improve FM in Kenya.

**Setting:**

Interviews were conducted at the different work sites of the participants, four of them in hospitals, one at a University and one in a hotel where a FM conference was held.

**Methods:**

An online questionnaire (response rate = 56%) and six semi-structured interviews were conducted amongst family physicians who completed their studies in Kenya. However, the focus was on the interviews.

**Results:**

Family physicians have different ideas of how FM should look like ideally, but all agree that family physicians should be team leaders of a primary healthcare team, taking care of a defined population. Lack of policies, low numbers of family physicians and the misunderstanding of FM by all stakeholders are the major challenges. Sixty-four percent of the participants work in rural areas, and 77% perceive their current work as FM.

**Conclusion:**

Family medicine must be defined and properly promoted. Various areas have been identified that require further research: assessing required number of family physicians, reasons and solutions for the low number of family physicians, funding possibilities, and research the most suitable definition of a Kenyan family physician.

## Introduction

Good primary healthcare can improve the health outcomes of patients at lower and more affordable costs.^1,2,3^ For this reason, Kenya, amongst other sub-Saharan African countries,^1,4,5,6,7^ has started to introduce family medicine (FM) to its health system.^2,8,9^ In Kenya, 80%^3^ of the 52.5 million inhabitants^10^ still live in the rural parts of the country,^3^ whilst only 16% of the Kenyan doctors are working in rural areas.^11^

FM is a medical discipline that is supposed to strengthen primary healthcare, especially in rural areas.^12,13^ According to the Kenyan FM Strategy, family physicians (FPs):

[*A*]re medical doctors providing competent clinical care over a wide range of patient conditions considering the patient’s physiologic, psychological, socio-economic, cultural and spiritual dimensions within the context of their family and community.^12^

In 2002, the Kenyan Association of FPs (KFP) was founded,^11^ the first Kenyan FM strategy was published in 2007 by the Kenyan Ministry of Health^12^ and from 2005 onwards, a total of five Kenyan universities started to train FPs. Currently (August 2019), there are 42 graduated FPs with 35 graduated from Moi, five from Aga Khan and two from Maseno University. However, more graduates are expected within the next months. The aim of the study was to establish the current state of FM in Kenya and identify areas for more research and actions to support and improve FM in Kenya.

## Methods

The study was conducted using face-to-face interviews in English with FPs who graduated from a Kenyan University and are now working within Kenya.

Prior to the interviews, a self-created online questionnaire was sent to 39 of the 42 FPs via email (three FPs plus one graduate from a Tanzanian University participated in the pre-test).

The questionnaire consisted of 17 questions. The aim was to identify FPs for the interviews and assess the current status of FM in Kenya.

Twenty-two FPs responded (response rate = 56%). Out of those, six FPs with different characteristics (gender, location of job, work experience in terms of time and content, job satisfaction) were interviewed using a semi-structured interview guide. Data saturation was achieved after the fourth interview.

The interview guide consisted of nine questions about the doctors’ current job, their view on FM, challenges and the current status of FM. The interviews took between 21 min and 1 h 8 min. The interviews were audio-recorded, transcribed and analysed by using a thematic content analysis approach.^14^

### Ethical consideration

Ethical clearance was obtained from the IREC committee of Moi University, Kenya (Ref: IREC/2019/134) and from FHMLREC of Maastricht University, the Netherlands (Ref: FHML/GH_2019.052).

## Results

### Questionnaires

The questionnaire showed that 2 (9%) of the 22 FPs are women, and 20 (91%) are men. Moreover, 17 (77%) FPs perceive their current job as FM and 18 (82%) have already been working in a field that is not FM at some point in their career.

Furthermore, 14 (64%) of the participants state that they are working in a rural area, 6 (27%) in urban areas and 2 (9%) in peri-urban areas. The 22 participants work in 16 counties within Kenya (see Table 1).

**TABLE 1:** Distribution of family physicians over counties.

County	Number of FPs
Baringo	1†
Bungoma	3
Embu	1
Kiambu	2
Kisumu	2
Machakos	2
Nairobi	3
Nakuru	1†
Nandi	1
Nyandarua	1
Tharaka-Nithi	1
Transnzoia	1
West Pokot	1
Muranga	1
Vihiga	1
Migori	1

FP, family physicians.

† , one person works in both counties.

### Interviews

All participants agreed that the main current challenge is the lack of common understanding of FM. Stakeholders such as doctors from other specialties, policy makers, the community and even FPs themselves either do not understand what FM is or have different understandings of it. Roles and responsibilities of a FP have to be defined, and based on that, policies need to be designed and implemented:

‘We don’t have clear policies that guide who a family physician is, colleagues and stakeholders have not understood what family medicine is all about. […] We as KFP need to define that role put it into policy and that’s what now we sell to the county governments as this is what our family physician does, this is where they are expected to be placed, and this is where you will end up having the biggest impact.’ (15MO04)

Being a FP in Kenya means doing something that no one has performed before in that context. In order to have the FM discipline be more recognised, FPs need to actually practice FM instead of hiding comfortably in other medical disciplines:

‘Of course, it’s cosy to stay in a ward and, you know, work there and have a defined schedule and stuff like that. […] But I still feel like there is much more that we can do [, *family physicians*] need to go out of their comfort zones and do what needs to be done. […] What is actually lacking is us ourselves to do what we are trained to do.’ (19MO06)

Funding FM is a problem, as there is not much money in preventative care:

‘Most of the money will be spent in the clinical curative science. Minimum of money spent on preventive aspects. […] If [*the family physicians*] know the curative medicine, you find them falling back in curative medicine because that is where they get more money.’ (08MO01)

Asking the question how FM should look like ideally and which role the FP plays, all participants agreed that FPs should be team leaders of primary healthcare teams who take care of defined populations:

‘[*T*]he family doctor should be a […] leader for a team of health workers who have been assigned a defined population. […] one family physician is working with a family health clinical officer, a family health nurse, […] a nutritionist, a social worker, […] a public health technician, with community health workers […]and they are assuring that [*the patients*] are optimally healthy. […] anybody who is sick, they already know the condition they have, and they are optimally taken care of.’ (08MO01)

Besides that, different views on what the role of an FP looks like in detail exist. One doctor, for instance, perceives FPs to be able to handle every case, no matter how complicated, whilst another doctor views himself as a gatekeeper who sees the patient first and, in case needed, refers to another specialist.

Two participants feel like they are already practicing FM the way it is supposed to be practiced. Both of them are in charge of a hospital, are seeing patients of all genders and age in the hospital, go to the communities where they treat patients for free if they cannot afford it and educate about preventative aspects:

‘For every month twice, I have to go to the community with a team of doctors, students, and nurses. We treat people for free. In the villages, in the marketplaces, in the church. We take advantage of any place where people might possibly meet. […] Now because they know the doctor is part of the village, he visits them, he talks with them, he goes to their local church, he brings medicine to them in the local market. They feel happy going to seek services from such a doctor.’ (10MO03)

## Discussion

Although FM has been defined by the Kenyan Ministry of Health, the main current challenge is the lack of understanding of how FM should look like in practice. Neither policy makers, patients and doctors of other medical disciplines, nor FPs themselves have a common understanding where to place an FP within the Kenyan health system. Therefore, one of the next major steps is to include all important stakeholders to write realisable FM policies. For doing so, approaches of FPs who already feel like they are practicing FM the way it is supposed to be practiced can be compared.

The low numbers of FPs have already been identified as a challenge in a study in 2012.^3^ Having been able to graduate only 42 FPs in 14 years is far too low. Further research should investigate the number of FPs needed, the reasons for the continuously low numbers of trainees, and come up with solutions to tackle the problem. Advocacy for FM could be one possible solution to it because doctors do not apply for the FM master’s programme if they do not know what it is.

As FPs are responsible for a defined population, no matter which socio-economic status, financing is a problem. In Kenya, only 19.59% of the population is health-ensured,^15^ meaning that over 42 million people do not have a health insurance. Family medicine should be accessible for everyone wherefore further research should investigate how FM can be funded sustainably and discuss the potential contributions of FM in achieving universal health coverage in Kenya.

Nevertheless, progress can be identified. The study in 2012^3^ revealed that filling gaps in other specialties is a problem. This is supported by the result that the majority of FPs already worked in other specialties at some point in their careers. Currently, however, three-quarters of the FPs are actually practicing FM. Furthermore, it is a great development that almost two-thirds are working in rural areas. Although one doctor more per county can hardly solve the health issues, it is also a progress that the 22 participants are distributed over 16 counties as it will help to introduce the new discipline into the entire country.

### Limitations

Because of the semi-structured interviews, the participants were free to set their own focus. Therefore, the results are biased because other FPs might have set a different focus. The online questionnaire had an animation mistake wherefore question 9 did not show up.

## Conclusion

Family medicine in Kenya is still in its infancy. The next most important step is to write realisable FM policies and do further research (see Box 1).

BOX 1 Areas that require further research.

- Asses the required number of family physicians that the Kenyan health system needs.
- Investigate the reasons for the continuously small remaining numbers of family medicine trainees and come up with solutions to tackle this problem.
- Investigate how family medicine can be funded sustainably and assess how universal health coverage can be a solution.
- Compare approaches of family physicians who feel like they are already practicing family medicine the way it is supposed to be practiced, in order to write suitable family medicine policies.

family medicine, family medicine.

BOX 2 Interview guide.

**Interview**

1. Can you give me a *short* overview about how your professional path looked like since your graduation?
2. How does your work look like at the moment: in which facility are you working and how does your daily work look like?
3. What is family medicine for you, how would you define it?
4. Do you see any challenges regarding family medicine? This can be anything from the training to the policy to the actual practice in the health facility.
5. In your opinion, how should family medicine in Kenya look like ideally? What exactly should be the role of a family physician?
6. What needs to change to achieve this?
7. In the questionnaire, many participants argued that family medicine lacks support and understanding by institutions such as the government, policy makers and so on. Can you say a few words about this?
8. In the questionnaire, it was also mentioned a few times that family medicine is not yet fully understood. What do you think about that statement? (Do you feel like there are even different understandings of what FM is between the Kenyan family physicians?)
9. If you take everything we discussed into consideration, could you summarise in *two or three sentences* how you see the current status of family medicine in Kenya?

FM, family medicine.

**TABLE 2:** Table of participants.

Participant	University of training	Year of graduation	Currently working in FM or not	County of current employment
19MA05	Maseno University	2019	FM	Bungoma
08MO01	Moi University	2008	Never worked in FM	Kiambu
10MO03	Moi University	2003	FM	Muranga
11MO02	Moi University	2002	FM	Kiambu
15MO04	Moi University	2004	FM	Baringo, Nakuru
19MO06	Moi University	2006	Not FM	Bungoma

FM, family medicine.
